# Multi-walled carbon nanotubes change morpho-functional and GABA characteristics of mouse cortical astrocytes

**DOI:** 10.1186/s12951-015-0152-y

**Published:** 2015-12-18

**Authors:** Joo-Ok Min, Seong Yeol Kim, Ueon Sang Shin, Bo-Eun Yoon

**Affiliations:** Department of Nanobiomedical Science and BK21 PLUS NBM Global Research Center for Regenerative Medicine, Dankook University, Dandae-ro, Dongnam-gu, Cheonan-si, Chungnam 330-714 Republic of Korea; Institute of Tissue Regeneration Engineering (ITREN), Dankook University, Dandae-ro, Dongnam-gu, Cheonan-si, Chungnam 330-714 Republic of Korea

**Keywords:** Multi-walled CNT, Astrocytes, GABA

## Abstract

**Background:**

Multi-walled carbon nanotubes (MW-CNTs) have been extensively explored for their possible beneficial use in the nervous system. CNTs have shown to modulate neuronal growth and electrical properties, but its effect that varying length of MW-CNTs on primary astrocyte roles have not been clearly demonstrated yet.

**Results:**

We investigate here the effect of MW-CNTs on astrocytic morphology, cell–cell interaction and the distribution of intracellular GABA (gamma-amino butyric acid). Primary cultured cortical astrocytes on MW-CNT-coated glass coverslips grow rounder and make more cell–cell interactions, with many cell processes, compared to astrocytes on poly-d-lysine (PDL) coverslips. In addition, intracellular GABA spreads into the cell processes of astrocytes on MW-CNT coverslips. When this GABA spreads into cell processes from the cell body GABA can be released more easily and in larger quantities compared to astrocytes on PDL coverslips.

**Conclusions:**

Our result confirm that MW-CNTs modulate astrocytic morphology, the distribution of astrocytic GABA, cell–cell interactions and the extension of cell processes. CNTs look to be a promising material for use neuroprosthetics such as brain-machine interface technologies.

**Electronic supplementary material:**

The online version of this article (doi:10.1186/s12951-015-0152-y) contains supplementary material, which is available to authorized users.

## Background

Nanotechnology is an emerging and fast growing field with, encouraging prospects that may revolutionize many disciplines such as engineering, biology, chemistry, physics and medicine [1]. Carbon nanotubes (CNTs) are hollow cylinders made of graphene sheets rolled in on themselves to form a tube [2]. They are categorized based on the number of carbon layers assembled together: single-walled (SW-CNTs), double-walled (DW-CNTs), and multi-walled (MW-CNTs) [3]. MW-CNTs consist of multiple concentrically rolled layers of graphene. Depending on the number of layers, the inner diameter of MW-CNTs varies from 0.4 nm to a few nanometers and the outer diameter varies characteristically from 2 nm to 20–30 nm [4]. The advantages of MW-CNTs over single-walled nanotubes include ease of mass production, a lower product cost per unit and a higher tensile strength [5, 6].

CNTs have been extensively explored for their beneficial use in nervous system tissue engineering, such as in CNT-based nerve scaffolds to drive nerve regeneration across a lesion site [2]. CNTs have shown much promise in neural applications [7, 8]. CNT planar strata were shown to modulate neuronal growth and neurite outgrowth in culture and can affect the electrical properties of neurons, effects that appear to sprout from electrical shortcuts, that is direct physical interactions between the CNTs embedded within the film and the neurons grown on it [9]. Indeed, over a narrow range of thicknesses, the CNT layers can affect the outgrowth of neurites, the number of growth cones, and the neuronal body area [10].

In addition, recent studies suggest that glia cooperate closely with neurons and actively participate in the regulation of synaptic transmission. Among the various types of glia, astrocytes make direct contact with neurons, forming tripartite synapses, where astrocytic processes are in close association with the presynapse and postsynapse at the synaptic junction [11, 12]. Astrocytes express diverse receptors for the corresponding neurotransmitters and release various gliotranmitters and neuroactive molecules [13–15].

It has been shown recently that effects of diverse nanostructures on glial cells. For instance, impaired autophagy flux in primary glial cells in Alzheimer’s disease mouse model promotes disease progression. Functionalized single-walled carbon nanotubes (SW-CNTs) restored normal autophagy by facilitating elimination of autophagic substrates [16]. SW-CNT-net can be used as growth substrate for astrocytes and label-freely detects local ATP release from these cells [17]. A number of studies demonstrated that CNTs are beneficial materials in neuron and neural stem cells, such as CNTs boost neuronal electrical signaling [18], direct stem cell differentication toward neural lineages [19] and control neurite outgrowth in vitro [20, 21]. Over a narrow range of thicknesses (10, 30, 60 nm) single-walled carbon nanotube films modulate the round morphology and increase the relative density of living cells [22]. However, the effect that varying length MW-CNTs on coated coverslips have on primary astrocytes is not understood. Therefore, we selected MW-CNTs of the most proliferative length (50 nm) [22, 23] and of those of long length (1000 nm) in order to investigate the effect of CNT. In this study, we evaluated MW-CNT cytotoxicity and cell–cell interaction. Furthermore, we investigated the distribution of astrocytic GABA, the major inhibitory neurotransmitter that has recently been reported to mediate tonic inhibition from glia [24].

## Methods

### Materials

Pristine MW-CNTs (>95 %, 15–20 nm outer diameter, 10–20 μm length, *p*-CNTs) were purchased from EMP (EM-Power Co., LTD, Korea). 3-aminopropyltriethoxysilane (APTES) and 1-ethyl-3-(3-dimethylaminopropyl) carbodiimide (EDC) were purchased from Sigma–Aldrich (USA). All supplementary chemicals were of analytical grades and used without further purification. Coverslips (12 mm diameter) were purchased from Marienfeld (Germany).

### Animals

P0-P2 Balb/c mice were used for primary astrocyte cultures. All experimental procedures described below were performed in accordance with the Dankook University Animal Experimentation Guidelines (Cheonan, Korea).

### Preparation of highly water-soluble multi-walled carbon nanotubes (MW-CNTs of lengths ~50 and ~1000 nm)

Highly water-soluble, carboxyl group functionalized multi-walled carbon nanotubes (MW-CNT-COOH of lengths ~50 and ~1000 nm (MW-CNT-50 and -1000) were prepared using the acid oxidative method and a homogenizer (NanoDeBee 45-3, Bee International). Typically, 2 g of pristine-CNTs (*p*-CNTs) were added to 100 mL of a concentrated 1:1 H_2_SO_4_/HNO_3_ solution and refluxed at 80 °C for 4 days. The mixture obtained was diluted with 100 mL of distilled water and then filtered through a 0.4 μm Millipore polycarbonate filter membrane. The resulting MW-CNT powders (average length ~1000 nm) were then continuously washed with distilled water until the filtrate pH reached 7. The MW-CNT-1000 obtained was further cut using the NanoDeBee process (5 cycles) to obtain MW-CNT-50 having a length of ~50 nm.

### Immobilization of MW-CNT-50 and MW-CNT-1000 on a glass substrate

**Fig. 1:**
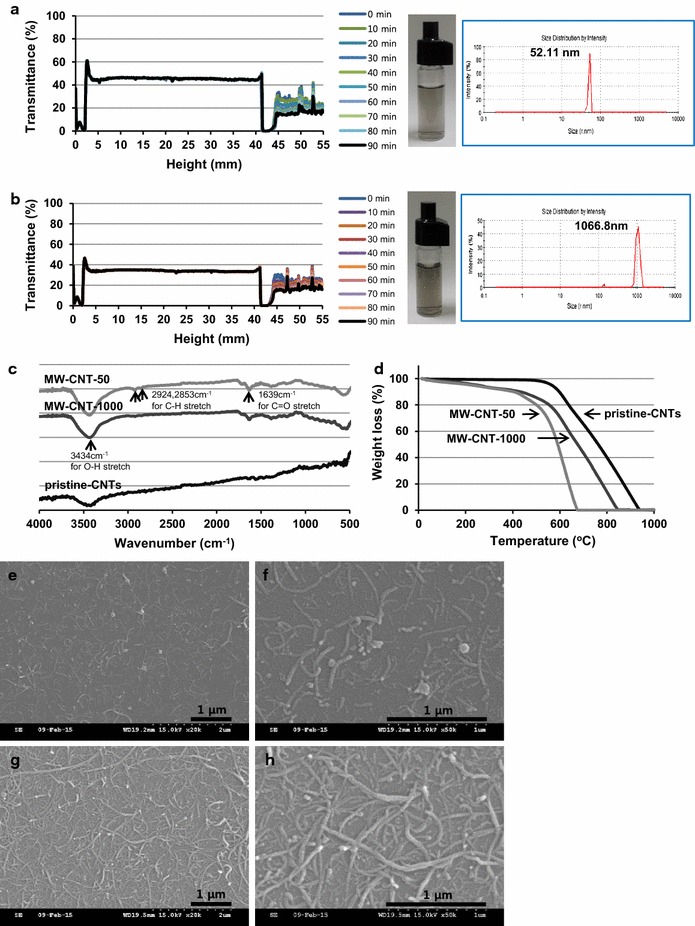
Characterization of MW-CNT-50 and MW-CNT-1000. **a**, **b** show dispersion stability of MW-CNT-50 (**a**) and MW-CNT-1000 (**b**) measured in water by Turbiscane Lab: Size distribution curves for MW-CNT-50 and MW-CNT-1000 and a photo of their aqueous solutions (5 × 10^−3^ mg/mL) homogeneously dispersed in water are shown as insets. **c**, **d** show characterizations of *p*-CNTs, MW-CNT-50, and MW-CNT-1000 by FT-IR (**c**) and TGA (**d**). **e**, **h** show SEM images of the surface morphology of coverslips uniformly coated with MW-CNTs; MW-CNT-50 (**e**, **f**) and MW-CNT-1000 (**g**, **h**); this SEM images with lower MW-CNT concentration in **e**, **f** were intentionally selected to prove that MW-CNT-50 molecules immobilized on the coverslip have an average length of 50 nm. The full scale SEM images of high magnification for MW-CNT-50 and MW-CNT-1000 samples were provided in Additional file 1: Figure S3 of the supplementary materials to show the similarity of the overall MW-CNT concentration for both samples

Prior to use, the glass (SiO_2_) substrates (coverslips) were ultrasonically cleaned in deionized water, acetone and ethanol. To prepare carbon nanotube layers on the pre-cleaned SiO_2_ substrates, the amine functionalized coverslips were initially treated with APTES. Coverslips were allowed to react with 10 w/v% of APTES in distilled water containing hydrochloric acid (pH = 3; adjusted with 2 N HCl) at 80 °C for 10 min. They were then rinsed with distilled water and dried at 110 °C. This process was repeated a further 4 times (totalling 5times). The amine functionalized coverslips (×5) were soaked in 1.5 mL of 0.1 w/v% aqueous CNT solution in the presence of EDC and HCl (pH = 5; adjusted with 1 N HCl) by shaking at room temperature (RT) for 3 h. They were then rinsed with distilled water and ethanol and let it dried in the air. The mono-layered CNT coating was confirmed using SEM microscopy as shown in Fig. 1e–h.

### Characterizations

The mean sizes of the prepared MW-CNTs were measured at room temperature (RT) with a Zeta sizer Nano ZS90 (Malvern, France) in aqueous solution and by field emission scanning electron microscopy (FE-SEM; MIRA II LMH, Tescan, Czech Republic). The surface morphologies of coverslips modified with the MW-CNT samples were also observed by FE-SEM. The specimens for FE-SEM analysis were subjected without coating with gold prior to examination. The dispersion stabilities of MW-CNT samples were tested using a Turbiscane Lab (Leanontech, France) in PBS solution at RT for about 1.5 h and measuring the backscattered light of a pulsed near infrared light source of wavelength 880 nm. Qualitative and quantitative analyses of MW-CNT samples were performed by Fourier-transform infrared (FT-IR) spectrometry and thermogravimetric analysis (TGA).

### Primary cortical astrocyte cultures

The cerebral cortex from P0 to P2 in postnatal Balb/c mice was dissected free of adherent meninges, minced and dissociated into a single-cell suspension by trituration. Then single-cell suspension cultured on coated poly-d-lysine culture dish. After 3 days, cell debris and medium were removed then fresh medium were added. 4 days after removing cell debris, cells were suspended using 0.05 % trypsin and cells were counted with a hematocytometer. Dissociated 2 $\times 10^{5}$ cells were plated onto 12-mm glass coverslips coated with 0.05 mg/ml multi-walled CNT or 0.1 mg/ml poly-d-lysine. Cells were grown in Dulbecco’s modified Eagle’s medium (DMEM) supplemented with 25 mM glucose, 10 % heat-inactivated horse serum, 10 % heat-inactivated fetal bovine serum, 2 mM glutamine, and 1 % penicillin–streptomycin. Cultures were maintained at 37 °C in a humidified atmosphere containing 5 % CO_2_.

### Scanning electron microscope

Primary cortical astrocytes are seeded on MW-CNT and PDL coverslips after 1 day and 4 days, after which the medium was removed and cells were washed once in 0.1 M phosphate buffered saline (PBS). After washing, astrocytes were fixed for 5 min in 2 % glutaraldehyde. For each coverslip type, cells were dehydrated for 5 min in 70 % ethanol and 90 % ethanol. Then cells were dehydrated twice in 100 % ethanol and incubated for 1 min in hexamethyldisilazane. Cells on each coverslip type were dried overnight at RT. After drying, astrocytes on both coverslip types were coated with platinum for 80 s. A series of images was obtained with a field emission scanning electron microscope (Hitachi). Astrocyte roundness was calculated using image J program. Roundness factor formula is 4π × [area (μm^2^)/[perimeter (μm^2^)].

### Measurement of cell viability and proliferation

Primary cortical astrocytes were seeded with the number of $2 \times 10^{5}$ cells for 1 day and 4 days and the cytotoxicity of MW-CNT compared to PDL coverslips was measured by adding. Cell Counting Kit-8 (CCK-8; Enzo life science, USA) solution and further incubating coverslips for 3 h. Optical density (OD) was then measured at absorbance wavelength 450 nm using a Versa Max microplate reader (Molecular Devices, USA). Rates of cell cytotoxicity and proliferation were calculated from the following equation. Cell viability = (ODsample − ODblank), where ODblank was obtained from the medium alone.

### Immunocytochemistry

Primary cortical astrocytes seeded on MW-CNT and PDL coverslips were fixed after 4 days in 4 % paraformaldehyde for 30 min at RT. After fixation, cells were washed three times in 0.1 M phosphate buffered saline (PBS). Cells were incubated for 1 h at RT with blocking solution (0.3 % Triton-X, 2 % normal serum in 0.1 M PBS). Then cells were incubated with primary antibody in 0.3 % Triton-X, 2 % normal serum in 0.1 M PBS, overnight at 4 °C on a shaker; Guinea pig anti-GABA antibody 1:400 (Millipore Bioscience Research), Chicken anti-GFAP antibody 1:500 (Millipore Bioscience Research). After washing three times in PBS, cells were incubated with the corresponding secondary antibodies; conjugated Alexa 647 Goat anti Guinea-pig antibody (1:200; Jackson ImmunoResearch Inc.), conjugated Alexa 488 Donkey anti-Chicken antibody (1:200; Jackson ImmunoResearch Inc.), for 2 h, followed by one rinse in PBS, and were then incubated one more time in DAPI (1:1000), followed by a further rinse in PBS. Then cells were mounted in fluorescent mounting medium. A series of fluorescence images was obtained with confocal microscopy (Zeiss, LSM 700) and images were processed for later analysis using ZEN 2010 imaging software. GFAP fluorescence intensity was measured using image J and Excel program. GFAP fluorescence intensity was measured following calculation (i.u.). The GFAP fluorescence intensity (i.u.) = cell intensity − (area × mean fluorescence of background).

### Results and discussion

To generate carbon nanotube layers on coverslips, highly water-soluble and carboxyl group functionalized multi-walled carbon nanotubes (~50 and ~1000 nm of length) were initially prepared (Step 1) and then the pre-cleaned SiO_2_ substrate were functionalized with amine groups via treatment with APTES (Step 2), followed by the immobilization of carboxyl group-functionalized CNTs through amide bond formation (Step 3). Physicochemical characterizations of the prepared MW-CNT samples (MW-CNT-50 and MW-CNT-1000) were made in terms of dispersion stability and average particle sizes in aqueous solution, functional groups, and thermal stability, using respectively Turbiscane Lab and Zeta sizer, FT-IR and TGA as shown in Fig. 1.

Unlike pristine CNTs (*p*-CNTs; see Addtional file 1: Figure S1), no noticeable changes in backscattered light fluxes and transmission were observed, meaning there was a clear surface wettability change from the hydrophobic charateristic of *p*-CNTs to the hydrophilic property of the modified MW-CNTs (Fig. 1a, b). The aqueous solutions of MW-CNT samples, which have some dark-color as shown in the inset photos of Fig. 1a, b, were highly homogeneous and nothing was deposited at the bottom of sample bottles even after a period of several days. Observations on the size changes of the prepared MW-CNT samples were conducted under aqueous conditions (1 × 10^−4^ wt%) by a Zeta sizer. As shown in Fig. 1 insets, the average sizes of MW-CNT samples were recorded to be, respectively, ~50 nm and ~1000 nm long. The measured average sizes in length could be confirmed in the SEM images as well, but generally it is hard to measure the accurate length in SEM images, because smaller MW-CNT pieces could be hidden under longer MW-CNT molecules which were congregated in each other (see Additional file 1: Figure S3).

An FT-IR study was performed to characterize the functional groups developed on MW-CNT surfaces (Fig. 1c). The functional groups (–O–H, –(C=O)–, and –COOH) as-formed after the functionalization of *p*-*CNTs* by chemical oxidation were observed clearly in the spectra of MW-CNT samples with average lengths of 50 and 1000 nm, for example, as strong bands at ~3434 cm^−1^ for the O–H stretch, 2924–2853 cm^−1^ for the –CH_2_– stretch, and 1725–1630 cm^−1^ for the C=O stretch, whereas the spectrum of *p*-CNTs showed very weak peaks in the same frequency ranges, demonstrating the intensive functionalization of the *p*-CNT surface with hydrophobic characteristics. Thermo gravimetric analysis (TGA: Fig. 1d) showed that both MW-CNT samples of ~50 and ~1000 nm length were slowly decomposed in the initial temperature range of <~425 °C, followed by an intensive secondary decomposition occurring in the temperature ranges of ~425 to ~670 °C for MW-CNT-50 and ~425 to ~840 °C for MW-CNT-1000, while the *p*-CNTs showed only an intensive decomposition curve witha steep slope in the temperature range of ~600 to ~930 °C. It was also clear that the gradual slope increase clearly occurred in accordance with the size reduction. Consequently, the resulting MW-CNTs with characteristics such as ~50 and ~1000 nm lengths and hydrophilic surfaces could be considered suitable for coating coverslips. After coating the surface of the coverslips with modified MW-CNTs, we examined the morphology changes of the functionalized MW-CNTs and the coverslip surface by SEM. A clear morphology change from aggregates of purchased long carbon nanotubes (about 17 μm in length; Additional file 1: Figure S2) to homogenously dispersed nanotubes is evident (Fig. 1e–h). Moreover, the rough surface of MW-CNTs coated on the surface of coverslips clearly indicates the oxidative damage of *p*-CNTs and the formation of hydrophilic functional groups such as -OH and -COOH on the MW-CNT surfaces, which may possibly encourage a chemical link such as an amide bond, between the glass coverslip and the MW-CNT surfaces and thus chemically bind MW-CNTs with the glass surface. In stark contrast, the smooth surface and the loose gathering of *p*-CNTs may indicate that only the possible π–π interaction between *p*-CNT surfaces that have few –OH and –COOH functional groups caused the heterogeneous agglomeration (Additional file 1: Figure S2). As shown in Fig. 1e–h, the highly uniform MW-CNT-coating in a monolayer on the coverslip may demonstrate that MW-CNT molecules are closely bound to the glass surface and that the smaller the MW-CNTs are, the more they are coated in monolayer.

**Fig. 2:**
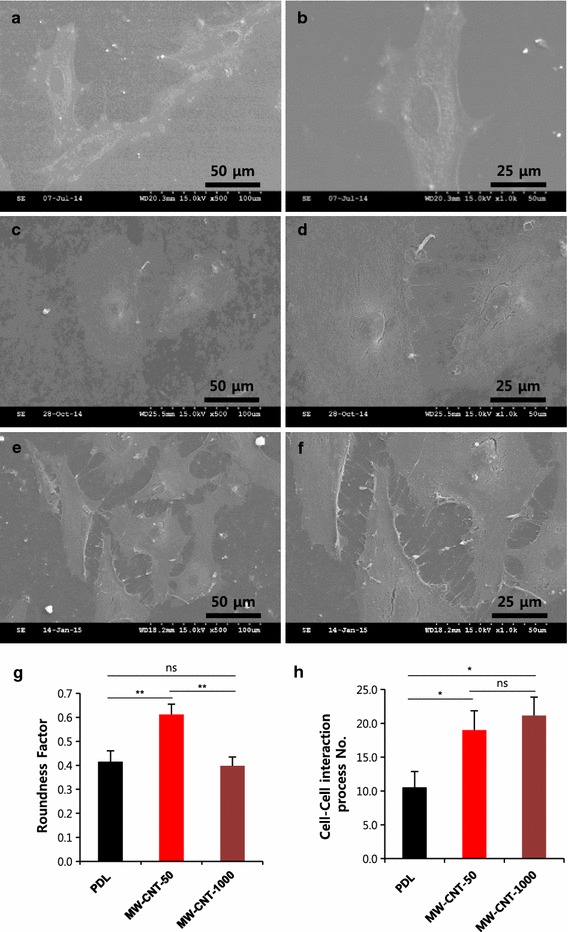
MW-CNTs affect astrocyte morphology as shown by scanning electron microscopy. **a**, **b** show primary cultured cortical astrocytes on PDL coverslips. **c**, **d** show astrocytes on MW-CNT-50 coverslips with much rounder cells. **e**, **f** show astrocytes on MW-CNT-1000, cell growth is similar to that seen on PDL. **g** shows roundness parameter graph. Astrocytes on MW-CNT-50 (n = 8) showed higher roundness factor compared to astrocytes on PDL (n = 10) and MW-CNT-1000 (n = 7). Roundness factor formula is 4π × [area (μm^2^)/[perimeter (μm^2^)]. **h** graph shows MW-CNTs (MW-CNT-50; n = 8, MW-CNT-1000; n = 7) make more cell–cell interactions, with many cell processes, compared to astrocytes on PDL coverslips (n = 10); (*p < 0.05), (**p < 0.01). Significant effects were determined by a one-way ANOVA analysis of variance

Based on observations from scanning electron microscopy, we assessed astrocyte morphology using morphometic parameters (Roundness factor). Roundness factor formula is 4π × [area (μm^2^)/[perimeter (μm^2^)]. The circularity of circle is 1, while thin thread is approximately 0. We found that astrocytes on MW-CNT-50 have a increased shape factor compared to astrocytes on PDL coverslips (Fig. 2a–d, g). Astrocytes on 1000 nm length MW-CNT coverslips were shown to have a similar shape to those on PDL coverslips (Fig. 2e–f, g). In addition, astrocytes can form gap junctions with neighboring cells, thereby forming interconnected groups of cells sharing a common cytoplasm [25]. Gap junction are classified as a type of intercellular junction with a function in intercellular communication [26, 27]. Therefore, we suggest that astrocytes on MW-CNTs (50, 1000 nm) have more cell–cell interaction, with higher numbers of cell processes compared to those seen on PDL coverslips (Fig. 2h). We determined that MW-CNT differential lengths change the morphology of astrocytes. In particular, MW-CNT-50 give a rounder shape than PDL, but they show more cell–cell interactions. A rounder shape does not thus decrease cell–cell interactions, but rather increases the number of cell processes.

**Fig. 3:**
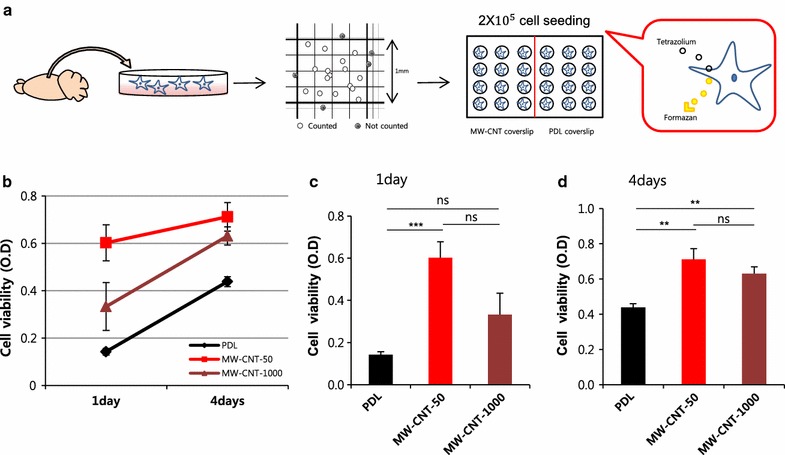
MW-CNTs are not cytotoxic and show more proliferation than PDL coverslips. **a** Scheme of measurement of primary cultured cortical astrocyte cytotoxicity. **b** Graph represents measurements of cytotoxicity of MW-CNT to astrocytes and cell proliferation compared to that on PDL coverslips. **c** *Graph* shows astrocytes after seeding for 1 day on MW-CNTs and PDL-coated coverslips. Astrocytes viability on MW-CNT-50 is higher than on PDL-coated coverslips. **d** Graph shows astrocytes after seeding for 4 days on MW-CNTs and PDL-coated coverslips. Astrocyte viability of MW-CNTs is higher than that on PDL-coated coverslips; PDL (n = 5), MW-CNT-50 (n = 5), MW-CNT-1000 (n = 5). Significant effects were determined by a one-way ANOVA analysis of variance; (*p < 0.05), (**p < 0.01), (***p < 0.001)

**Fig. 4:**
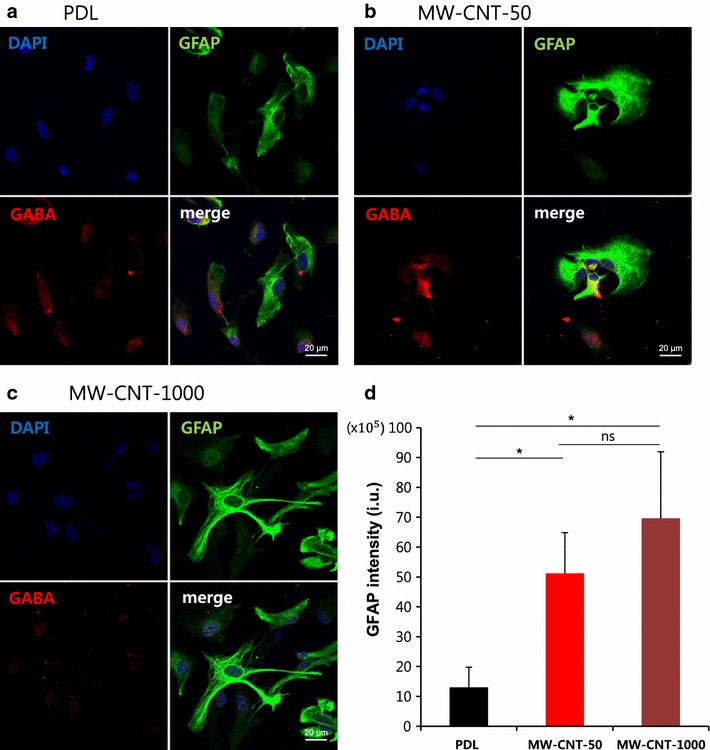
Astrocytic GABA on MW-CNTs spreads into cell processes. **a**–**c** Immunostaining of GABA using anti-GABA (*red*), anti-GFAP (*green*) antibody in primary cortical astrocytes on MW-CNT and PDL coverslips. **a** shows distribution of astrocytic GABA near the nucleus. **b** shows astrocytes on MW-CNT-50 to have a rounder shape compared to those on PDL and **c** shows distinct cell–cell interactions on MW-CNT-1000. **a**–**c** shows that intracellular GABA on MW-CNT has spread into cell processes (**b**, **c**) compared to the PDL coverslip (**a**); *Scale bar* 20 μm. **d** *Graph* shows MW-CNTs increase GFAP immunoreactivity compared to PDL (*p < 0.05); PDL (n = 7), MW-CNT-50 (n = 7), MW-CNT-1000 (n = 7). Fluorescence intensity (i.u.) = cell intensity − (area × mean fluorescence of background)

To date there is very little known about the potential neurotoxic effects of CNTs. Recent experiments in rat and fish showed that nanosized carbon particles can be taken up by olfactory neurons in the nose and are translocated to the brain, which would seem to make neurotoxicity of CNTs an important issue [28, 29]. Therefore, we evaluated the cytotoxicity of MW-CNTs and the proliferation of primary cortical astrocytes using CCK-8 solution in which viable cells convert WST (water-soluble tetrazolium salt) to formazan using dehydrogenase. WST receives two electrons from viable cells to generate a yellow or orange formazan dye. Formazan was released into the medium and then we measured optical density (Fig. 3a). We measured cell viability and proliferation, after seeding for 1 day and for 4 days. The 1 day period gives an initial astrocyte adhesion on MW-CNT (50, 1000 nm) and PDL coverslips and the 4 day period allows for proliferation of astrocytes seeded on MW-CNT and PDL coverslips. Cytotoxicity of MW-CNT-50 and MW-CNT-1000 is not different from astrocytes on PDL coverslips (Fig. 3b–d). After seeding for 1 day, the cell viability of astrocytes on MW-CNT-50 was increased and the relative density of live cells on MW-CNT-50 was fourfold that of cells on PDL. After seeding for 4 days, most of the higher cell viability was observed on the MW-CNT-50, while most of the proliferation ratio was measured on MW-CNT-1000 (Fig. 3d). Next, we investigated intracellular GABA distribution on MW-CNT and PDL coverslips. In MW-CNTs, astrocytic GABA spreads into more cell processes than occurs on PDL coverslips, and astrocytes on MW-CNT-50 have a rounder shape, implying that this rounder shape does not affect GABA distribution (Fig. 4). When astrocytic GABA spreads into cell processes from the cell body, GABA can be released more easily and in greater quantities compared to that from astrocytes on PDL coverslips. The diffusional GABA into cell processes accelerates release to tripartite synapses and increases astrocyte-neuron interactions, implying increased bidirectional communication because proteins such as transporters, receptors and channels are involved in neurotransmitter release. In particular, and consistent with SEM imaging, MW-CNT-1000 makes more cell–cell interactions (Fig. 4c). Some reports show that the cause of neurodegenerative diseases, such as Parkinson’s disease (PD) and Alzheimer’s disease (AD), is a progressive loss of structure or function of neurons, as well as neuronal cell death. Also, increased GFAP immunoreactivity decreases tissue damage and neuronal loss and demyelination [30, 31]. Based on our immunocytochemical data, MW-CNTs showed increased GFAP immunoreactivity compared to astrocytes on PDL (Fig. 4). Electrical stimulation neural activity is the basis of a number of technologies for the restoration of sensory or motor functions [32–35], brain-machine interfaces (BMI) [36, 37], deep brain stimulation therapies for neurological disorders [38], such as Parkinson’s disease and depression. Metal electrodes are inadequate prospects for the miniaturization needed to attain neuronal-scale stimulation and recording because of their poor electrochemical properties, high stiffness, and propensity to fail due to bending fatigue. However, tissue contact impedance of CNT fibers is remarkably lower than that of state of the art metal electrodes, making them suitable for recording single-neuron activity [39]. Therefore, increased GFAP immunoreactivity on MW-CNTs have possibility for new therapy of neurodegenerative disorders and MW-CNTs can be a promise material for biocompatibility.

## Conclusion

We expect that MW-CNTs of diverse length could be used efficiently in brain-machine interface technologies, for delivery of drugs to the brain in brain disease therapy because increased cell–cell interaction may reflect active communications between astrocytes and neurons. A recent study showed that single-walled CNTs modulate the morpho-functional and proliferative characteristics of astrocytes [22]. We confirmed that MW-CNTs also modulate intracellular morphology and proliferative characteristics such as an increased cell–cell interaction and the modulation of GABA distribution. CNTs look to be a promising material for use in varied fields, particularly in, brain-machine interface technologies, as they have been shown to efficiently stimulate neurons and have long-term stability, while eliciting a significantly reduced inflammatory response in Parkinsonian rodents [39]. Further studies are required to demonstrate astrocyte-neuron interactions with MW-CNTs, which may generate further advances in current brain disease therapy.

## Additional file

10.1186/s12951-015-0152-y Supplementary figures.

## Abbreviations

MW-CNTs: multi-walled carbon nanotubes
PDL: poly-d-lysine
GABA: gamma-amino butyric acid
