# Supplementary material for: Multi-walled carbon nanotubes change morpho-functional and GABA characteristics of mouse cortical astrocytes
Source: J Nanobiotechnology. 2015 Dec 18;13:92. doi: 10.1186/s12951-015-0152-y (PMC4683728; doi:10.1186/s12951-015-0152-y)
Supplement: Supplementary file 1 — 10.1186/s12951-015-0152-y Supplementary figures. [file 12951_2015_152_MOESM1_ESM.docx]

**Supplementary Materials for**

**Multi-walled carbon nanotubes change morpho-functional and GABA characteristics of mouse cortical astrocytes**

Joo-Ok Min^1,a^ , Seong Yeol Kim^1,2,a^, Ueon Sang Shin^1,2,＊^, Bo-Eun Yoon^1,＊^

^1^Department of Nanobiomedical Science & BK21 PLUS NBM Global Research Center for Regenerative Medicine, Dankook University, Dandae-ro, Dongnam-gu, Cheonan-si, Chungnam, 330-714, Republic of Korea

^2^Institute of Tissue Regeneration Engineering (ITREN), Dankook University, Dandae-ro, Dongnam-gu, Cheonan-si, Chungnam, 330-714, Republic of Korea

^a^These authors contributed equally to this work.

^*^Corresponding authors

**Fig. S1** **Dispersion stability of pristine-CNTs measured by Turbiscane Lab analyzer**

Inset photo of the aqueous solution (5x10^-3^ mg/mL) shows pristine-CNTs deposited at the bottom of the glass bottle.


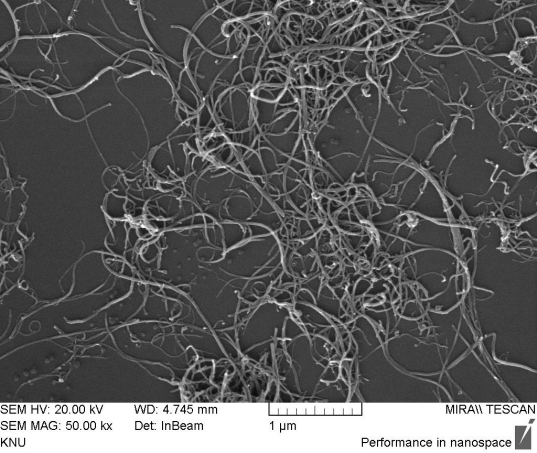


**Fig. S2** **SEM image of pristine carbon nanotubes (*p*-CNTs)**

Pristine-CNTs have smooth surfaces and display loose gathering.


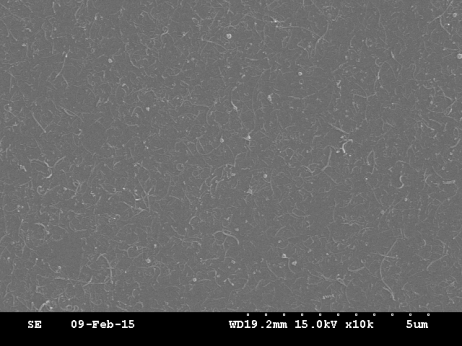

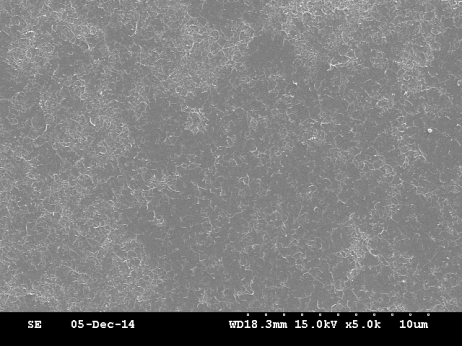

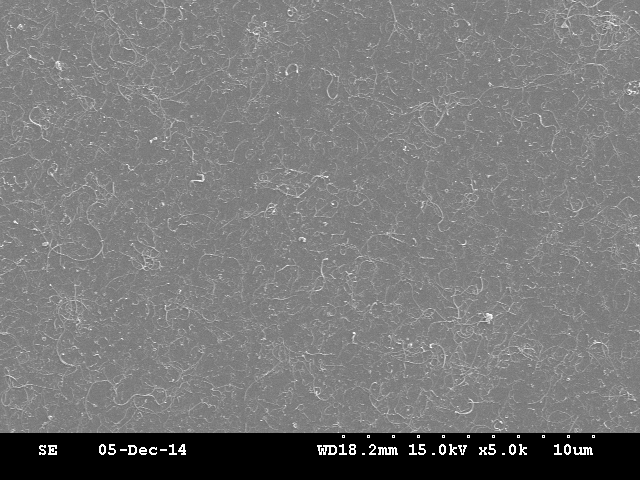

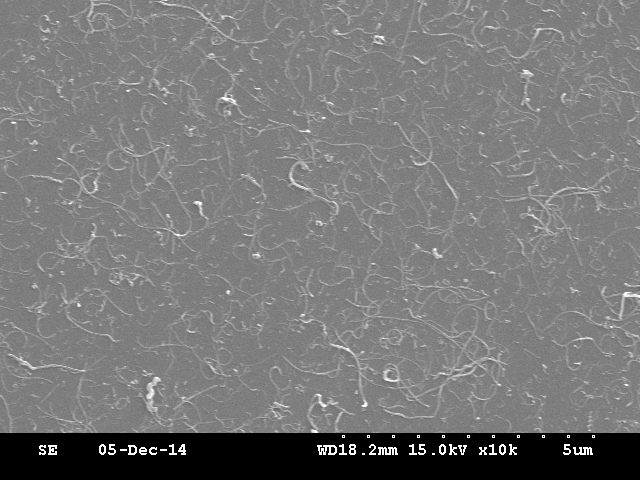


**(A)**

**Average 1000 nm MW-CNTs**

**Average 50 nm MW-CNTs**

**(C)**

**(B)**

**(D)**

**Fig. S3 Full scale SEM images of low (A and C) and high (B and D) magnification for MW-CNT-50 (A and B) and MW-CNT-1000 (C and D) samples.** These images clearly demonstrate the similarity of the overall MW-CNT concentration and the difference in MW-CNT length for the two type of samples.
